# Supplementary material for: Pretravel plans and discrepant trip experiences among travelers attending a tertiary care centre family travel medicine clinic
Source: PLoS One. 2022 Feb 3;17(2):e0262075. doi: 10.1371/journal.pone.0262075 (PMC8812894; doi:10.1371/journal.pone.0262075)
Supplement: S1 File — (PDF) [file pone.0262075.s001.pdf]

SickKids Family Travel Clinic

Pre-Travel Health Consultation and History Form

Personal Information: Please complete this section Date:

Traveler's Name:

Date of Birth: Male [ ] Female [ ]

Address:
____
____

Telephone: (Home): ____________________ (E-mail): ________________________
(Work): ____________________ (cell): ________________________

Occupation:

Country of Birth: Citizenship:

Trip Information:

Date of Departure from home: Return date/length of trip:

Do you intend to travel frequently in the future? [ ] Yes [ ] No [ ] Maybe
Have you traveled internationally in the past?[ ] Yes [ ] No Where?
____
____
____
____

Itinerary: Please give **ALL** countries and the **cities/regions** to be visited, **including stopovers, duration of each stay, and in the order** (if possible) to be visited:

1.____
2.____
3.____
4.____
5.____
6.____
7.____

Destination: (Check all that apply) Urban [ ] Rural [ ] Remote [ ] At High Altitude [ ] Beach [ ] Jungle/Forest [ ]

Is this a fixed itinerary? Yes [ ] No [ ] Unsure [ ]

Purpose of trip: (check all that apply)

Vacation [ ] Medical care [ ] Business [ ]
Education/Study [ ] Adoption [ ] Volunteer/Humanitarian [ ]
Visiting Friends and/or Relatives [ ] Long-stay traveler [ ]
Cruise [ ] Religious/Pilgrimage [ ] Other: ____________________________

Organized tour? Yes [ ] No [ ] Partly [ ] Tour Group Name: ______________________
Explain: ____

Accommodations: 4-5 Star Hotel [ ] 2-3 Star Hotel [ ] Hostel [ ]
Staying with locals/family/friends [ ]
Rented House/Apt [ ] Camping [ ] Cruise Ship/Boat [ ]
Safari [ ] Other: ______________________________________

Will you be travelling alone? Yes [ ] No [ ]

If no, Explain
____
____

Planned Activities: (check all that apply)

Air Travel [ ] Biking [ ] Hiking [ ] Snorkeling [ ]
Swimming: Freshwater [ ] Salt Water [ ] Rafting [ ] Boating [ ] Scuba [ ]
Climbing/Trekking [ ] Contact with Animals [ ] Cave/spelunking [ ]
Public Transport [ ] (bus, train, etc) Visiting schools, hospitals or orphanages [ ] Motorcycle/Scooter [ ]

Other:
____
____

Have you obtained travel medical insurance? Yes [ ] No [ ]
Details:

Health History:

Health Care Provider: Telephone:

Address:

Do you have any chronic health problems for which you take medication on a regular basis or see a health care provider?
Yes [ ] No [ ]

If yes, please explain:
____
____
____
____

Are you currently under the care of a physician for any health problem: Yes [ ] No [ ]

If yes, please explain:
____
____
____
____

Immunization History

Immunization	YES	Date(s) Received	NO	Unsure	Had the Disease
Tetanus- Diptheria Vaccine/Tdap (IM)					
Measles, Mumps, Rubella (SC)					
Typhoid (IM)					
Typhoid Oral (PO)					
Influenza (IM)					
Hepatitis A- 2 doses (IM)					
Hepatitis B- 3 doses (IM)					
Twinrix (IM)					
Polio- childhood series					
Polio- adult dose or booster					
Chicken pox/Varicella (IM/SC)					
Meningococcal – e.g., Menomune, Menactra (IM)					
Rabies (IM)					
Japanese Encephalitis (SC)					
Yellow Fever (SC)					
Pneumococcal – e.g., PPV, Prevnar (IM/SC)					
Other-please list:					

Health History, cont'd.:

Do you currently have or have a past history of:

Antidepressant or psychiatric medication use_______________ Yes [ ] No [ ]
Depression, anxiety, panic attacks_______________ Yes [ ] No [ ]
Psoriasis (skin disease)_______________ Yes [ ] No [ ]
Seizures or convulsions_______________ Yes [ ] No [ ]
Cardiac conduction defect, have a pacemaker_______________ Yes [ ] No [ ]
Diabetes _______________ Yes [ ] No [ ]
Heart disease or surgery_______________ Yes [ ] No [ ]
Respiratory (lung) disease_______________ Yes [ ] No [ ]
Muscle or bone problems_______________ Yes [ ] No [ ]
Intestinal problems including heartburn or reflux_______________ Yes [ ] No [ ]
Immune disorder (chemotherapy, HIV, bone marrow or organ transplant,
Rheumatoid arthritis treatment)_______________ Yes [ ] No [ ]
Live/work closely with anyone with immune disorder _______________ Yes [ ] No [ ]
Thymus gland surgery or disorder (myasthenia gravis, DiGeorge syndrome) ____ Yes [ ] No [ ]
History of altitude illness_______________ Yes [ ] No [ ]
Surgery or hospitalization in last 5 years_______________ Yes [ ] No [ ]
Have you had any transfusions or blood products in the past 5 years?_________ Yes [ ] No [ ]
Have you ever had Hepatitis (liver infection)?_______________ Yes [ ] No [ ]
Has your spleen been removed?_______________ Yes [ ] No [ ]
Do you drink alcohol regularly?_______________ Yes [ ] No [ ]
Do you smoke?_______________ Yes [ ] No [ ]
Have you ever had a TB test?_______________ Yes [ ] No [ ]
If yes, Result: ___ mm. CXR: Normal [ ] Abnormal [ ]
History of tendonitis / Achille's heel rupture_______________ Yes [ ] No [ ]
Other medical problem_______________ Yes [ ] No [ ]

Please explain any "yes" answers:
____
____
____

Allergies:

Medication(s) Yes [ ] No [ ] If yes, list: ______________________
Reaction to vaccine Yes [ ] No [ ] If yes, list: ______________________
Egg or other food allergies Yes [ ] No [ ] If yes, list: ______________________
Environmental Yes [ ] No [ ] If yes, list: ______________________
(Pollens, dust, hay fever, asthma, Iodine, etc.)
Animals Yes [ ] No [ ] If yes, list: ______________________
Bee stings Yes [ ] No [ ]
Have you ever experienced anaphylaxis (severe allergic reaction)?_____ Yes [ ] No [ ]
Describe: ____

Medications:
Please list **<u>all</u> prescribed and over-the-counter medications and supplements** you use:

Medication or supplement:	Reason for use:
1	
2	
3	
4	
5	

Others Comments:
____
____
____

Women:
When was your last menstrual period? ______________ Was it normal? Yes [ ] No [ ]
Are you currently or are you trying to become pregnant? Yes [ ] No [ ]
Any risk of an unplanned pregnancy? Yes [ ] No [ ]
Are you breastfeeding? Yes [ ] No [ ] Formula? Yes [ ] No [ ]
What form of contraception do you use? ______________________________

Please tell us any additional information that you believe is important for us to know as you prepare for your current trip:
____
____
____
____
____

Pre-Travel Prescriptions: To be completed by Clinician

Prescriptions	Dose	Quantity	Refills

Pre-Travel Counseling (Check all that apply): To be completed by Clinician

	Food and Water
	Med Care Abroad
	Fresh Water Risks
	Water Treatment
	TD Self-Care
	Travel Insurance
	Water Sports
	Alcohol and Drug Use
	Insect Protection
	Medical Kits
	Climate Risks
	Travel Stress
	Malaria ABCD
	Altitude Illness
	HIV PEP
	STD/ HIV
	Dengue
	Rabies Prevention and Post-Bite Care
	Personal Safety and Security
	Accidents
	Pregnancy Issues
	Post-Trip Issues
	Personal Medications
	Chronic Disease Care During Travel
	Other (please specify):

Notes (To be completed by Clinician):

I have answered this questionnaire fully and to the best of my ability.

Traveler’s signature ____________________________________ Relationship if minor ______________ Date ________________

Reviewed by: ____________________________________ RN/ NP/MD

Consent

I hereby acknowledge that the risks and benefits of the immunizing agent(s), tests and/or drugs given to be today have been explained to me and I hereby give my consent to treatment (or the treatment of my child)

Signature Date

SickKids Employees Only

Please note that all documentation related to care provided by the SickKids Family Travel Clinic will be entered into your Occupational Health file. By signing below, I consent to the sharing of my personal health information (PHIPA) between the staff of Occupational Health and the staff of the Travel Clinic.

I understand that this consent is in effect from the date indicated below, until I expressly revoke it.

☐ I give my consent

☐ I do not give my consent
Reason:

Signature	Printed Name	Employee#	Date
