# Supplementary material for: Pretravel plans and discrepant trip experiences among travelers attending a tertiary care centre family travel medicine clinic
Source: PLoS One. 2022 Feb 3;17(2):e0262075. doi: 10.1371/journal.pone.0262075 (PMC8812894; doi:10.1371/journal.pone.0262075)
Supplement: S2 File — (PDF) [file pone.0262075.s002.pdf]

Prescreen

Record ID ________________________________

Family Unique Number ________________________________

Respondent Unique ID ________________________________

Email Address ________________________________

Actual Occurrence of Risk

The goal of this questionnaire is to understand how your actual trip itinerary differed from the trip itinerary you described in your pre-travel assessment form. Through this questionnaire, we hope to understand how trip itinerary commonly changes, and use this information to improve pre-travel care provided to international travelers.

Any information you give will remain strictly confidential and will be available only to direct members of the study team.

No individual level data will be reported.

This questionnaire should take approximately 5 minutes to complete.

We thank you for your time.

1. a) What was your departure date? ______________________________
(Leave blank if unknown)

1. b) What was your return date? ______________________________
(Leave blank if unknown)

2. How many countries did you visit on your recent trip? (including stopover countries)
◯ 1
◯ 2
◯ 3
◯ 4
◯ 5
◯ 6
◯ 7
◯ 8
◯ 9
◯ 10
◯ >10

i. What was the first country you visited? ______________________________

How long was your stay? ______________________________
(Number of days)

Did you visit any cities in the first country?
◯ Yes
◯ No

Which cities did you visit?

____

How long did you spend in each city?

____
(Number of days)

Did you visit any rural area(s) in the first country?
◯ Yes
◯ No

Describe the rural area(s) you visited. __________________________________

How long did you spend in each area? __________________________________
(Number of days)

ii. What was the second country you visited? ____________________________

How long was your stay? ____________________________
(Number of days)

Did you visit any cities in the second country?
- ◯ Yes
- ◯ No

Which cities did you visit? __________________________________

How long did you spend in each city? __________________________________
(Number of days)

Did you visit any rural area(s) in the second country?
- ◯ Yes
- ◯ No

Describe the rural area(s) you visited. __________________________________

How long did you spend in each area? __________________________________
(Number of days)

iii. What was the third country you visited? ____________________________

How long was your stay? ____________________________
(Number of days)

Did you visit any cities in the third country?
- ◯ Yes
- ◯ No

Which cities did you visit? __________________________________

How long did you spend in each city? __________________________________
(Number of days)

Did you visit any rural area(s) in the third country?
- ◯ Yes
- ◯ No

Describe the rural area(s) you visited. __________________________________

How long did you spend in each area?

____
(Number of days)

iv. What was the fourth country you visited? ________________________

How long was your stay? ________________________
(Number of days)

Did you visit any cities in the fourth country?
- ◯ Yes
- ◯ No

Which cities did you visit?

____

How long did you spend in each city?

____
(Number of days)

Did you visit any rural area(s) in the forth country?
- ◯ Yes
- ◯ No

Describe the rural area(s) you visited.

____

How long did you spend in each area?

____
(Number of days)

v. What was the fifth country you visited? ________________________

How long was your stay? ________________________
(Number of days)

Did you visit any cities in the fifth country?
- ◯ Yes
- ◯ No

Which cities did you visit?

____

How long did you spend in each city?

____
(Number of days)

Did you visit any rural area(s) in the fifth country?
- ◯ Yes
- ◯ No

Describe the rural area(s) you visited.

____

How long did you spend in each area?

____
(Number of days)

vi. What was the sixth country you visited? ________________________

How long was your stay? ______________________ (Number of days)

Did you visit any cities? ◯ Yes ◯ No

Which cities did you visit? ______________________

How long did you spend in each city? ______________________ (Number of days)

Did you visit any rural area(s) in the sixth country? ◯ Yes ◯ No

Describe the rural area(s) you visited. ______________________

How long did you spend in each area? ______________________ (Number of days)

vii. What was the seventh country you visited? ______________________

How long was your stay? ______________________ (Number of days)

Did you visit any cities in the seventh country? ◯ Yes ◯ No

Which cities did you visit? ______________________

How long did you spend in each city? ______________________ (Number of days)

Did you visit any rural area(s) in the seventh country? ◯ Yes ◯ No

Describe the rural area(s) you visited. ______________________

How long did you spend in each area? ______________________ (Number of days)

viii. What was the eighth country you visited? ______________________

How long was your stay? ______________________ (Number of days)

Did you visit any cities in the eighth country? ◯ Yes ◯ No

Which cities did you visit? __________________________________

How long did you spend in each city? __________________________________ (Number of days)

Did you visit any rural area(s) in the eighth country?
◯ Yes
◯ No

Describe the rural area(s) you visited. __________________________________

How long did you spend in each area? __________________________________ (Number of days)

ix. What was the ninth country you visited? ____________________________

How long was your stay? ____________________________ (Number of days)

Did you visit any cities in the ninth country?
◯ Yes
◯ No

Which cities did you visit? __________________________________

How long did you spend in each city? __________________________________ (Number of days)

Did you visit any rural area(s) in the ninth country?
◯ Yes
◯ No

Describe the rural area(s) you visited. __________________________________

How long did you spend in each area? __________________________________ (Number of days)

x. What was the tenth country you visited? ____________________________

How long was your stay? ____________________________ (Number of days)

Did you visit any cities in the tenth country?
◯ Yes
◯ No

Which cities did you visit? __________________________________

How long did you spend in each city? ______________________ (Number of days)

Did you visit any rural area(s) in the tenth country?
◯ Yes
◯ No

Describe the rural area(s) you visited. ______________________

How long did you spend in each area? ______________________ (Number of days)

xi. What were the additional countries you visited? ______________________

How long was your stay? ______________________ (Number of days)

Did you visit any cities in these countries?
◯ Yes
◯ No

Which cities did you visit? ______________________

How long did you spend in each city? ______________________

Did you visit any rural area(s) in these countries?
◯ Yes
◯ No

How long did you spend in each area? ______________________

Describe the rural area(s) you visited. ______________________

3. Did you visit any remote areas?
◯ Yes
◯ No
(a remote area is any region that is more than 80 km or 1 hour transport from a major regional hospital)

How long did you spend in the remote area(s)? ______________________ (Number of days)

Describe the remote area(s) you visited. ______________________

4. Did you visit any high altitude areas (higher than 3000 meters/10 000 feet above above sea level)?
◯ Yes
◯ No

How long did you spend in the high altitude area? ______________________ (Number of days)

Where was the high altitude area? __________________________________

5. Did you visit any beaches?
   - ◯ Yes
   - ◯ No

How long did you spend at the beaches? ______________________________
(Number of days)

Where were the beaches? __________________________________

6. Did you visit any forests or jungles?
   - ◯ Forest
   - ◯ Jungle
   - ◯ Both
   - ◯ No

How long were you there for? ______________________________
(Number of days)

Describe the area(s) you visited. __________________________________

7. On your trip, did you stay at:
   - ☐ 4-5 star hotel
   - ☐ 2-3 star hotel
   - ☐ Hostel
   - ☐ With locals/family/friends
   - ☐ Rented house/apartment
   - ☐ Camping
   - ☐ Cruise ship/boat
   - ☐ Safari
   - ☐ Other

   (check all that apply)

If other, please specify ______________________________

8. On your trip, did you do any of the following:
   - ☐ Air travel
   - ☐ Biking
   - ☐ Hiking
   - ☐ Snorkeling
   - ☐ Freshwater swimming
   - ☐ Saltwater swimming
   - ☐ Rafting
   - ☐ Boating
   - ☐ Climbing/trekking
   - ☐ Have contact with animals
   - ☐ Caving/spelunking
   - ☐ Scuba diving
   - ☐ Use public transport (bus, train, etc)
   - ☐ Visit schools, hospitals, or orphanage
   - ☐ Motorcycle/scooter

   (check all that apply)

9. On your trip, did you consume alcohol?
   - ◯ Yes
   - ◯ No

What kind of alcohol did you consume?
   - ☐ Beer
   - ☐ Wine
   - ☐ Liquor/spirits

   (check all that apply)

How many drinks of beer did you have per week? ______________________________

How many drinks of wine did you have per week? ______________________________

How many drinks of liquor/spirits did you have per week? ______________________________

10. On your trip, did you use any recreational drugs?
    ◯ Yes
    ◯ No

What recreational drug(s) did you use?

____

11. On your trip, did you get any tattoos or piercings?
    ◯ Yes
    ◯ No

12. On your trip, did you have sex with a new partner?
    ◯ Yes
    ◯ No

13. On your trip, did you have unprotected sex?
    ◯ Yes
    ◯ No
